# Between session reliability of heel-to-toe progression measurements in the stance phase of gait

**DOI:** 10.1371/journal.pone.0200436

**Published:** 2018-07-12

**Authors:** Vanessa Ade, Dale Schalkwijk, Michael Psarakis, Michael D. Laporte, Timothy J. Faras, Remi Sandoval, Fadi Najjar, Peter W. Stubbs

**Affiliations:** 1 Discipline of Physiotherapy, Department of Health Professions, Faculty of Medicine and Health Sciences, Macquarie University, Sydney, Australia; 2 Faculty of Health Sciences, Australian Catholic University, Sydney, Australia; 3 Hammel Neurorehabilitation and Research Center, Aarhus University, Hammel, Denmark; 4 Discipline of Physiotherapy, Graduate School of Health, University of Technology Sydney, Sydney, Australia; Northwestern University, UNITED STATES

## Abstract

The objective of the current study was to determine the test-retest reliability of heel-to-toe progression measures in the stance phase of gait using intraclass correlation coefficient (ICC) analysis. It has been proposed that heel-to-toe progression could be used as a functional measure of ankle muscle contracture/weakness in clinical populations. This was the first study to investigate the test-retest reliability of this measure. Eighteen healthy subjects walked over the GAITRite® mat three times at a comfortable speed on two sessions (≥ 48 hours apart). The reliability of the heel-to-toe progression measures; heel-contact time, mid-stance time and propulsive time were assessed. Also assessed were basic temporal-spatial parameters; velocity, cadence, stride length, step length, stride width, single and double leg support time. Reliability was determined using the ICC_(3,1)_ model and, fixed and proportional biases, and measures of variability were assessed. Basic gait temporal-spatial parameters were not different between sessions (*p* > 0.05) and had excellent reliability (ICC_(3,1)_ range: 0.871–0.953) indicating that subjects walked similarly between sessions. Measurement of heel-to-toe progression variables were not different between sessions (*p* > 0.05) and had excellent reliability (ICC_(3,1)_ range: 0.845–0.926). However, these were less precise and more variable than the measurement of standard temporal-spatial gait variables. As the current study was performed on healthy populations, it represents the ‘best case’ scenario. The increased variability and reduced precision of heel-to-toe progression measurements should be considered if being used in clinical populations.

## Introduction

The stance phase of normal gait is characterized by heel-contact, foot progression (where the tibia progresses over the foot) and push off [1]. Psarakis et al. [2] described these phases as ‘heel-to-toe progression’ (heel-contact, mid-stance and propulsive phases) and developed a method, using a pressure sensitive walkway, to measure the time taken for each phase. It was suggested that heel-contact time (which begins when the heel touches the mat and ends when the toe touches the mat), and mid-stance time (which begins when the toes touches the mat and ends when the heel leaves the mat) could be used to functionally characterise ankle flexor weakness and/or ankle extensor contracture [2]. That study [2] found that patients with multiple sclerosis with ankle extensor contracture and ankle flexor weakness had a reduced heel-contact time and increased mid-stance time on the more affected leg compared to the less affected leg and healthy controls.

Ankle weakness and contracture are usually measured statically (i.e. while sitting or lying) and not during walking. When measured statically, ankle flexor strength and ankle extensor range of motion are valid and reliable [3–5]. However, the impairment, and resulting activity limitation during walking, can only be inferred from static measurements which may not be a true functional representation of the impairment. Clinically, during walking, these impairments are often quantified using clinician observation, which is poorly to moderately reliable [6,7]. Therefore, an objective method of measuring ankle weakness and contracture during walking that can be performed quickly and easily would be useful.

The measurement of heel-to-toe progression has been investigated in patients with multiple sclerosis and healthy subjects, however the reliability and variability of the measurement was not reported [2]. To be a worthwhile measurement, that could be used clinically, the measure needs to be reliable. Although pressure sensitive mats are easy to use and reliable for most temporal-spatial measurements [8–14], the reliability can vary (i.e. is lower for base of support [8]). The purpose of the current study was to assess the reliability of heel-to-toe progression in healthy people on two sessions spaced ≥ 48 hours apart. If the measure is reliable in healthy people, further investigation of the measure in clinical populations could be warranted.

## Methods

Eighteen healthy subjects participated in the study (14 males, 4 females; age: 27 ± 4 years (mean ± SD); height: 1.80 ± .09 m; weight: 80 ± 10 kg). Fifteen subjects were right leg dominant. Included subjects were > 18 years with no recent history of musculoskeletal or neurological injuries. Ethical approval was attained from the Macquarie University Human Research Ethics Committee (approval number: 5201600533) and adhered to the Declaration of Helsinki. All subjects provided written informed consent.

### Experimental procedures

Measurement of temporal-spatial gait parameters were performed on the GAITRite® portable walkway system (overall length: 9 m, active area: 8 m, CIR Systems Inc. Franklin, NJ). Subjects walked 13 meters with 2.5 meters at the start and end of the active area.

Subjects were tested twice with ≥ 48 hours between sessions (4 ± 2 days apart). Prior to testing, subjects performed a warm-up by walking on a treadmill at a comfortable speed (4.8 ± 0.5 km/h). The same speed was used for day 1 and day 2. Following the warm up, the treadmill was stopped, subjects waited for 30 seconds on the treadmill, stepped off the treadmill and waited another 10 seconds before performing three passes over the GAITRite mat. During GAITRite walking, subjects walked at a comfortable speed and focused on a target placed at eye level at the end of the mat.

### Outcome measurements

The primary outcome measure was the reliability of heel-to-toe progression during stance; the heel-contact phase, mid-stance phase and propulsive phase [2]. These variables were calculated from heel-on time, toe-on time, heel-off time and toe-off time. The heel-contact phase was calculated by subtracting toe-on time by heel-contact time. The mid-stance phase was calculated by subtracting the heel-off time with the toe-on time. The propulsive phase was calculated by subtracting toe off time by heel-off time.

Secondary outcome measures were speed, cadence, stride length, step length, stride width, single and double leg support time.

### Data analysis

Partial footsteps were excluded as only complete footsteps could be used for analysis. These occurred at the beginning or end of the active area. Variables were determined from the direct output generated by the GAITRite software.

Reliability was assessed between days for each variable. Single leg measures were analysed separately for the dominant and non-dominant legs.

For each variable, the absolute agreement using an ICC_(3,1)_ model was calculated [15,16]. As the same protocol was performed on day 1 and day 2, and the same rater was used on both days, an ICC_(3,1)_ model was appropriate [9,16]. Interpretation of the ICC values were: excellent reliability (ICC > 0.75), fair to good reliability (ICC 0.40–0.75) and poor reliability (ICC < 0.40) [9,12]. Bland-Altman plots were used to visualise the data. The mean difference between day 1 and day 2 (fixed bias) was assessed using paired t-tests. Significant paired t-tests indicated a fixed bias due to day. Proportional bias was assessed, for each variable, by fitting a linear regression to the data from Bland-Altman plots (mean of day 1 and day 2 with the difference between day 1 and day 2 for each subject). Significant R^2^ values indicated proportional bias. The Standard Error of Measurement (SEm) was calculated for each variable as detailed by Atkinson and Nevill [17]. The Coefficient of Variation (COV) was calculated for each variable, day and subject by expressing the standard deviation of the individual step measurements as a percentage of the mean [17]. Significance was set to *p* < 0.05. Data were analysed using MATLAB (version R2017b). Statistics were performed using SPSS (version 21).

## Results

The test-retest reliability (ICC_(3,1)_) was significant (*p* <0.001) and excellent for all variables with ICC’s ranging from 0.845 to 0.953 (Table 1). There was no significant difference between day 1 and day 2 (fixed bias) for any measured variable (*p* > 0.05, Table 1). There was proportional bias for heel-contact on the dominant leg (*p* = 0.019, R^2^: 0.30) but not any other variable (*p* > 0.05). The SEm (Table 1) and the within subject COV for each day and each variable (S1 Table) were generally higher for measures of heel-to-toe progression than most standard temporal-spatial gait measurements.

**Table 1:** The spatial and temporal measurements for each variable on day 1 and day 2, Standard Error of Measurement (SEm), mean difference between day 1 and day 2 (fixed bias), and the test-retest reliability.

	day 1 mean (SD)	day 2 mean (SD)	mean difference (95% CI)	SEm	reliability ICC_(3,1)_ (95% CI)
velocity, cm/s	147.9 (17.2)	150.2 (17.2)	-2.3 (-6.4 to 1.8)	5.9	0.881 (0.717 to 0.954)
step count, number	26.3 (2.4)	26.1 (2.7)	0.2 (-0.4 to 0.9)	0.7	0.871 (0.691 to 0.949)
cadence, steps/min	113.1 (7.3)	113.5 (8.2)	-0.4 (-1.8 to 1.1)	2.0	0.931 (0.827 to 0.974)
stride length, cm	156.9 (14.0)	158.7 (12.6)	-1.8 (-4.5 to 0.9)	3.9	0.911 (0.782 to 0.966)
stride width, cm	10.7 (2.3)	10.8 (2.0)	-0.1 (-0.5 to 0.3)	0.6	0.930 (0.824 to 0.973)
*dominant leg*					
step length, cm	78.6 (7.3)	79.4 (6.6)	-0.8 (-2.2 to 0.6)	2.0	0.915 (0.792 to 0.967)
single support time, ms	406.5 (24.9)	405.8 (25.8)	0.7 (-3.2 to 4.7)	5.4	0.953 (0.879 to 0.982)
double support time, ms	250.7 (37.0)	248.9 (43.0)	1.7 (-7.4 to 10.9)	9.1	0.899 (0.752 to 0.961)
heel-contact time, ms	148.2 (34.6)	150.9 (43.8)	-2.7 (-11.2 to 5.9)	11.8	0.909 (0.776 to 0.965)
mid-stance time, ms	237.4 (67.8)	235.1 (71.7)	2.3 (-11.4 to 16.1)	18.7	0.926 (0.813 to 0.971)
propulsive time, ms	271.3 (40.7)	269.6 (38.7)	1.7 (-7.5 to 10.8)	12.5	0.898 (0.749 to 0.961)
*non-dominant leg*					
step length, cm	78.3 (6.9)	79.3 (6.2)	-1.0 (-2.4 to 0.3)	2.0	0.903 (0.759 to 0.962)
single support time, ms	409.0 (26.9)	408.4 (27.8)	0.6 (-3.8 to 5.0)	6.0	0.951 (0.874 to 0.981)
double support time, ms	250.8 (37.3)	249.3 (44.3)	1.5 (-8.1 to 11.1)	9.5	0.893 (0.738 to 0.959)
heel-contact time, ms	161.0 (33.9)	165.4 (38.7)	-4.4 (-11.6 to 2.8)	10.3	0.918 (0.798 to 0.968)
mid-stance time, ms	240.4 (69.1)	232.9 (66.4)	7.5 (-8.8 to 23.7)	22.8	0.884 (0.721 to 0.954)
propulsive time, ms	258.6 (35.6)	258.9 (35.5)	-0.3 (-10.4 to 9.8)	13.8	0.845 (0.631 to 0.939)

## Discussion

The current study demonstrates that the measure of heel-to-toe progression has excellent reliability between sessions in healthy subjects. Despite this, it is more variable than standard gait measurements (using the GAITRite system) with the SEm and COV being generally higher for measures of heel-to-toe progression. The implications of this will be discussed below.

Similarly to previous studies [8], the test-retest reliability of standard gait measurements was excellent. As shown in the current study, the reliability of heel-to-toe progression measures was also excellent. Despite this, for heel-contact, mid-stance and propulsive times, the COV was high for heel-contact (~25%), mid-stance time (~19%), and propulsive time (~10%). Other than stride width (~14%), these COVs were consistently higher than standard gait measurements (supplementary material). In addition, the SEm was higher for measures of heel-to-toe progression indicating that these may not be as precise as standard gait measurements (Table 1). As the COV and SEm indicate less precise and more variable estimates, this needs to be considered when using these measurements in clinical populations. The population in the current study were young and healthy and represent the ‘best case’ scenario. Therefore, in clinical populations with (potential) gait instability, the variability and precision of the measurements maybe higher compared to current values.

To date, one study has used this measure to describe heel-to-toe progression in clinical populations [2]. That study determined that heel-contact time increased and mid-stance time decreased in the more affected side of patients with multiple sclerosis, with ankle extensor contracture and ankle flexor weakness. The current study shows that heel-contact time and mid-stance time were the most variable of the measured variables. The reason for this could be that the movement from heel-contact to mid-stance is unstable or it could indicate the lack of sensitivity of the GAITRite mat to detect a relatively stable phenomenon. As the balance of heel-contact and mid-stance is particularly important in assessing drop foot, with a reduction in heel-contact time and increase in mid-stance time in these patients, the increased COV and SEm observed in the current study, and its effect on measurement values, needs to be considered.

One benefit of using this measure of heel-to-toe progression is that it is an objective functional measure that can be easily performed clinically. Currently, the most valid and reliable methods to determine heel-to-toe progression are biomechanical analysis using gait analysis software. These require long set-up times that can be difficult and time consuming to perform and analyse. An easier alternative is to visually observe heel-to-toe progression. Although cheap and easy to perform, is moderately to poorly reliable to assess the movement, and changes in the movement, over time [6,7]. Clinically used static measures (in sitting or lying) to measure ankle range of motion and strength, although valid and reliable [3–5], do not provide the extent or the effect of the movement during walking. Therefore, as this measure of heel-to-toe progression is reliable (although variable), it could be more clinically useful than currently used measurements to measure strength and range of motion of the ankle.

### Limitations

The current study was performed on healthy subjects. The reason for this was to attain a ‘best-case’ reliability estimate and to assess the measurement in people in whom the measurement would unlikely change between days. The reliability would likely be lower between sessions in clinical populations as is observed in other temporal-spatial walking parameters [12]. Secondly, in our protocol, subjects performed a warm up at matched speeds. This may not occur in clinical or natural settings and may result in an overestimation of the reliability. This was done to ensure that participants were in a controlled state prior to walking on the GAITRite between days.

There was a proportional bias for the heel-contact time on the dominant leg. As the average (of day 1 and day 2) heel-contact time increased, the difference between the heel-contact time of day 1 and day 2 became greater. This was the only parameter that showed proportional bias and it is difficult to ascertain why. Given that the R^2^ value was 0.3 and (in total) 17 tests for proportional bias were performed, with no other variable showing proportional bias, we believe it is likely type 1 statistical error.

### Conclusion

The measure of heel-to-toe progression and its sub parts, heel-contact time, mid-stance time and propulsion time, was reliable in young healthy subjects. Despite this, the measure heel-contact time and mid-stance time were more variable than standard GAITRite measures. The implications of this are unknown but should be considered, especially in clinical populations. Given that measures of strength and contracture are usually measured in lying or sitting, this measurement could provide a quantitative approach to assess the success of strength and range of motion exercises in clinical populations during walking.

## Supporting information

S1 Table The coefficient of variation for each variable, for each subject on each day. (XLSX)

S1 File The subject characteristics and data associated with the manuscript. (XLSX)

## References

[1] Perry J, Burnfield J. Gait analysis: normal and pathological function. 2nd Edition Thorofare, New Jersey: SLACK Incorporated; 2010.

[2] Psarakis M, Greene D, Moresi M, Baker M, Stubbs P, Brodie M, et al Impaired heel to toe progression during gait is related to reduced ankle range of motion in people with Multiple Sclerosis. Clin Biomech. 2017; 49: 96–100. 10.1016/j.clinbiomech.2017.08.012 28898816

[3] Moseley A, Adams R. Measurement of passive ankle dorsiflexion. Aust J Physiother. 1991; 37: 175–181. 10.1016/S0004-9514(14)60540-7 25026482

[4] Diong J, Harvey L, Kwah LK, Clarke JL, Bilston LE, Gandevia SC, et al Gastrocnemius muscle contracture after spinal cord injury: a longitudinal study. Am J Phys Med Rehabil. 2012; 91: 1–10. 10.1097/PHM.0b013e318274605a 23117273

[5] Kwah LK, Herbert RD, Harvey LA, Diong J, Clarke JL, Martin JH, et al Passive mechanical properties of gastrocnemius muscles of people with ankle contracture after stroke. Arch Phys Med Rehabil. 2012; 93: 1185–1190. 10.1016/j.apmr.2012.02.009 22502803

[6] Krebs DE, Edelstein JE, Fishman S. Reliability of observational kinematic gait analysis. Phys Ther. 1985; 65: 1027–1033. 3892553 10.1093/ptj/65.7.1027

[7] Coutts F. Gait analysis in the therapeutic environment. Man Ther. 1999; 4: 2–10. 10463015 10.1016/s1356-689x(99)80003-4

[8] Van Uden CJT, Besser MP. Test-retest reliability of temporal and spatial gait characteristics measured with an instrumented walkway system (GAITRite®). BMC Musculoskelet Disord. 2004; 5: 13 10.1186/1471-2474-5-13 15147583 PMC420245

[9] Menz HB, Latt MD, Tiedemann A, Kwan MMS, Lord SR. Reliability of the GAITRite® walkway system for the quantification of temporo-spatial parameters of gait in young and older people. Gait Posture. 2004; 20: 20–25. 10.1016/S0966-6362(03)00068-7 15196515

[10] Webster KE, Wittwer JE, Feller JA. Validity of the GAITRite™ walkway system for the measurement of averaged and individual step parameters of gait. Gait Posture. 2005; 22: 317–321. 10.1016/j.gaitpost.2004.10.005 16274913

[11] McDonough AL, Batavia M, Chen FC, Kwon S, Ziai J. The validity and reliability of the GAITRite system’s measurements: A preliminary evaluation. Arch Phys Med Rehabil. 2001; 82: 419–425. 10.1053/apmr.2001.19778 11245768

[12] Kuys SS, Brauer SG, Ada L. Test-retest reliability of the GAITRite system in people with stroke undergoing rehabilitation. Disabil Rehabil. 2011; 33: 1848–1853. 10.3109/09638288.2010.549895 21265611

[13] Bilney B, Morris M, Webster K. Concurrent related validity of the GAITRite walkway system for quantification of the spatial and temporal parameters of gait. Gait Posture. 2003; 17: 68–74. 12535728 10.1016/s0966-6362(02)00053-x

[14] Beckwée D, Degelaen M, Eggermont M, Gonzalez-Rodriguez M, Lefeber N, Vaes P, et al Validity and test-retest reliability of the Stride Analyzer in people with knee osteoarthritis. Gait Posture; 2016; 49: 155–158. 10.1016/j.gaitpost.2016.06.039 27423404

[15] Portney LG, Watkins MP. Statistical measures of reliability. Foundations of clinical research Applications to practice 2nd ed Upper Saddle River, New Jersey: Prentice Hall Health; 2000 p. 557–586.

[16] Shrout PE, Fleiss JL. Intraclass correlations: Uses in assessing rater reliability. Psychol Bull. 1979; 86: 420–428. 18839484 10.1037//0033-2909.86.2.420

[17] Atkinson G, Nevill AM. Statistical methods for assessing measurement error (reliability) in variables relevant to sports medicine. Sport Med. 1998; 26: 217–238. 10.2165/00007256-199826040-00002 9820922

